# Syndromic surveillance of abortions in beef cattle based on the prospective analysis of spatio-temporal variations of calvings

**DOI:** 10.1038/srep18285

**Published:** 2015-12-21

**Authors:** A. Bronner, E. Morignat, G. Fournié, T. Vergne, J-L Vinard, E. Gay, D. Calavas

**Affiliations:** 1 ANSES-Lyon, Epidemiology Unit, Lyon, France; 2 Royal Veterinary College, Hatfield, Hertfordshire, UK

## Abstract

Our objective was to study the ability of a syndromic surveillance system to identify spatio-temporal clusters of drops in the number of calvings among beef cows during the Bluetongue epizootic of 2007 and 2008, based on calving seasons. France was partitioned into 300 iso-populated units, *i.e.* units with quite the same number of beef cattle. Only 1% of clusters were unlikely to be related to Bluetongue. Clusters were detected during the calving season of primary infection by Bluetongue in 28% (n = 23) of the units first infected in 2007, and in 87% (n = 184) of the units first infected in 2008. In units in which a first cluster was detected over their calving season of primary infection, Bluetongue was detected more rapidly after the start of the calving season and its prevalence was higher than in other units. We believe that this type of syndromic surveillance system could improve the surveillance of abortive events in French cattle. Besides, our approach should be used to develop syndromic surveillance systems for other diseases and purposes, and in other settings, to avoid “false” alarms due to isolated events and homogenize the ability to detect abnormal variations of indicator amongst iso-populated units.

According to European regulations, Member States must implement clinical surveillance of bovine brucellosis either to document their brucellosis-free status or to detect outbreaks in order to control the disease in the country. France has been recognized officially free of the disease since 2005, and the event-driven (i.e. “passive” or “clinical”) surveillance system relies on the mandatory notification and testing of each aborting cow. However, under-reporting is high^1^. In addition, this type of surveillance is brucellosis-specific, whereas many others diseases can cause abortions^2^,^3^,^4^,^5^. In this context, there is a need to improve bovine abortion surveillance by exploring other ways to detect disease events.

One of these ways is syndromic surveillance, which can be defined as the real-time (or near real-time) monitoring of one or several non-specific health indicators^6^. These indicators are usually designed based on data collected for purposes other than surveillance and, where possible, automatically generated so as not to impose an additional burden on the data providers^6^. Their variations are studied over time or space-and-time, and a statistical alarm is raised if abnormal variations are detected (*i.e.* above the random variations).

Calving data are reported by animal owners for traceability purposes. According to Regulation (EC) No. 1760/2000 of the European parliament, each farmer has to notify every birth on the farm. These data are centralized in a National Cattle Register that each Member State must maintain. With regard to bovine abortions, our hypothesis was that in the event that an abortive disease is introduced, the increase in abortions would be followed by a decrease in the number of calvings at the time and location in which the aborted cows should have calved.

Accordingly, our aim was to study the feasibility of implementing syndromic surveillance based on calving data to identify and locate abortive events in beef cattle, a population in which current abortion surveillance is particularly weak^1^. A straightforward approach using spatial information involves studying the time-series variations of the indicator separately within each spatial unit, and setting a statistical “alarm” representing an abnormal variation of the indicator over time in a given unit^7^,^8^. However, this approach ignores the possible dependence of variations of the indicator amongst units, and is therefore unrealistic. Another approach, which we chose and detailed below, is to look for spatio-temporal clusters of abnormal variation of the indicator, using scan statistics^9^,^10^,^11^. The Bluetongue epizootic that spread across France in 2007 and 2008 and increased the risk of abortions in cattle^12^,^13^,^14^ was used to illustrate this methodology. Considering the significant seasonal variations in calvings among beef cows, drops were expected at the end of the calving seasons during which Bluetongue spread.

By mimicking a prospective analysis of the spatio-temporal variations in calving data aggregated into spatial units, our objective was to study the capacity of a syndromic surveillance system to identify spatio-temporal clusters of drops in the number of calvings during the Bluetongue epizootic of 2007 and 2008, based on calving seasons.

## Results

### Calving seasons and Bluetongue exposure per unit

Population size was null over at least one week of the study period in seven units. These units were excluded because the prediction uncertainty would have been too important. Thus, the study focused on 293 iso-populated units (98%). Among these units, the median value of the population size per unit was 17,178 cow-days with a coefficient of variation equal to 0.3.

**Figure 1:**
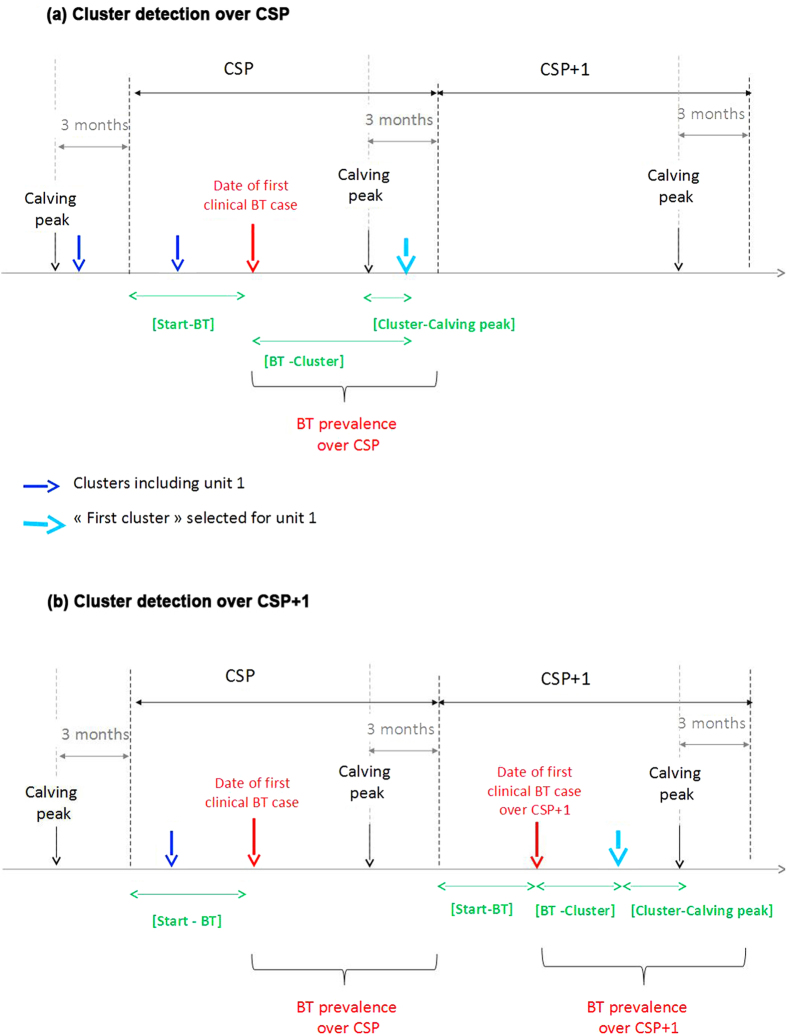
Illustrations of the method used to define calving seasons and their characteristics. Unit 1 (Fig. 1a) is a unit in which the first cluster was detected over CSP (the calving season of primary infection by Bluetongue), which was therefore the only calving season studied. Unit 2 (Fig. 1b) is a unit in which the first cluster was detected over CSP+1 (the calving season that follows CSP), and both CSP and CSP+1 were studied. For each unit and each calving season, we computed the time elapsed between the start of this calving season and the first clinical Bluetongue (BT) case ([Start-BT]), and the Bluetongue prevalence. By focusing on calving seasons over which the first cluster was detected (CSP for unit 1, CSP+1 for unit 2), we calculated the time elapsed between the first clinical Bluetongue case and the first cluster detection ([BT-cluster]), and between calving peak and the first cluster detection ([Cluster-Calving peak]).

**Figure 2:**
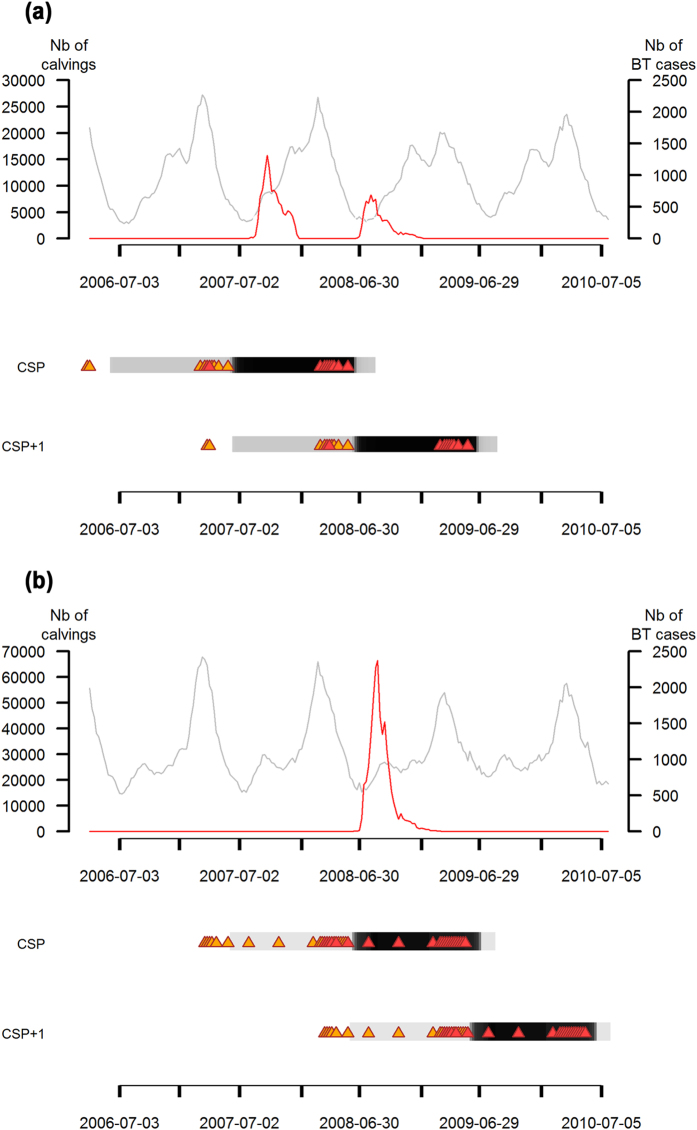
Description of calving peaks and calving seasons selected for each unit over time. Fig. 2a focuses on all units first infected by Bluetongue (BT) in 2007 and ((Fig. 2b)) on all units first infected by Bluetongue in 2008. For each figure, the upper graph displays the weekly variations in the total number of calvings (in grey) and the weekly variations in the number of clinical Bluetongue cases (in red). The lower graph refers to the distribution of CSP (the calving season of primary infection by Bluetongue) and CSP+1 (the calving season that follows CSP) defined for each unit: the colour over one week is related to the number of calving seasons (defined for each unit) that include this week (the darker the colour, the higher this number). Triangles refer to the weeks of calving peaks used to define the beginning (in orange) and the end (in red) of calving seasons.

Clinical Bluetongue cases were reported in all units, enabling the identification in each unit of a calving season during which the Bluetongue infection was reported for the first time. This calving season will hereafter be referred to as the calving season of primary infection (CSP) (Fig. 1a,b). Bluetongue was first detected in 2007 in 82 units and in 2008 in 211 units. Clinical Bluetongue cases were also reported over the calving season that follows CSP (referred to CSP+1 hereafter) in all units first infected in 2007 and in 15 (7%) out the 211 units first infected in 2008 (Fig. 1a,b). Each calving season lasted 52 weeks. The period from 30 July 2007 to 9 June 2008 (*i.e.* 45 weeks) was included in the CSP for 94% (n = 77) of the units first infected by Bluetongue in 2007. The period from 14 July 2008 to 25 May 2009 (i.e. 45 weeks) was included in the CSP for 86% (n = 181) of the units first infected by Bluetongue in 2008 (Fig. 2a,b).

### Sensitivity and specificity

**Figure 3:**
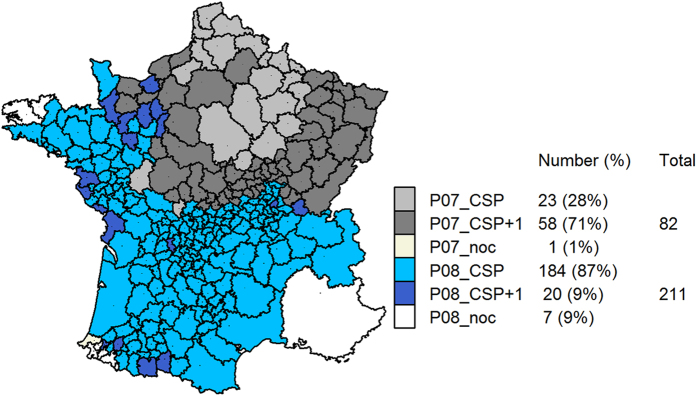
Spatial distribution of units according to their periods of primary infection and first cluster detection. This map was created using R^35^. It displays the 300 iso-populated units into which France was partitioned, and their population weighted centroid (black points). Note that the population density per unit is inversely proportional to the size of the units. The legend presents the number of units and the proportion of units first infected by Bluetongue in 2007 (n = 82) and 2008 (n = 211), respectively. *P07_CSP*, *P07_CSP*+*1* and *P07_noc* units refer to units first infected by Bluetongue in 2007 in which a cluster was detected over CSP, CSP+1, or in which no cluster was detected, respectively. *P08_CSP*, *P08_CSP*+*1* and *P08_noc* units refer to units first infected by Bluetongue in 2008 in which a cluster was detected over CSP, CSP+1, or in which no cluster was detected, respectively.

Setting the likelihood ratio (LLR) threshold at 350 offered the best compromise between sensitivity and specificity of the surveillance system. Among clusters with an LLR above 350, five false positive clusters that included between 10 and 12 units were identified (out of 388 clusters, i.e. 1% of clusters). A cluster was detected after their first clinical Bluetongue case in 81 (99%) units first infected by Bluetongue in 2007 and 204 (97%) units first infected by Bluetongue in 2008. The spatial distribution of units according to the calving season over which the first cluster was detected, and their number, are displayed in Fig. 3.

**Figure 4:**
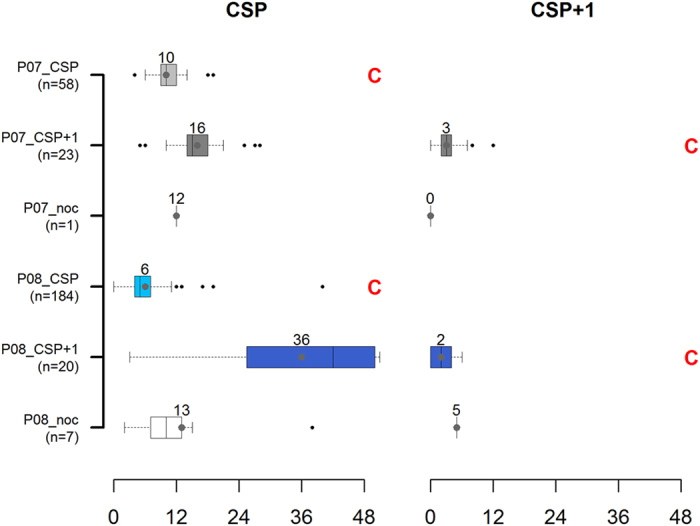
Distribution of the time elapsed between the beginning of each calving season and the first clinical Bluetongue case. This figure displays the distribution of the time elapsed between the beginning of the calving season and the first clinical Bluetongue case (in weeks) over CSP (the calving season of primary infection by Bluetongue, on the left) and CSP+1 (the calving season that follows CSP, on the right) among units, depending on the date of first Bluetongue infection and of first cluster detection. *P07_CSP*, *P07_CSP*+*1* and *P07_noc* units refer to units first infected by Bluetongue in 2007 in which a cluster was detected over CSP, CSP+1, or in which no cluster was detected, respectively. *P08_CSP*, *P08_CSP*+*1* and *P08_noc* units refer to units first infected by Bluetongue in 2008 in which a cluster was detected over CSP, CSP+1, or in which no cluster was detected, respectively. Calving seasons over which a cluster was detected are identified by a red letter C. Numbers refer to the average period (in weeks).

For the units first infected in 2007, Bluetongue prevalence was significantly higher in the units where the first cluster was detected during their CSP as compared to the units where the first cluster was detected during their CSP+1 or where no cluster was detected (both p-values < 2.10^−16^); similar results were obtained for those units first infected in 2008 (both p-values < 2.10^−16^). Also, for the units first infected in 2007, the time elapsed between the start of CSP and the first clinical Bluetongue case (left plot in Fig. 4) was significantly lower in the units where the first cluster was detected during their CSP as compared to the units where the first cluster was detected during their CSP+1 (p-value < 2.10^−6^); similarly in the units first infected in 2008, this delay was significantly lower in the units where the first cluster was detected during their CSP as compared to the units where the first cluster was detected during their CSP+1 or where no cluster was detected (p-values of 2.10^−6^ and 0.006, respectively).

### Early cluster detection

**Figure 5:**
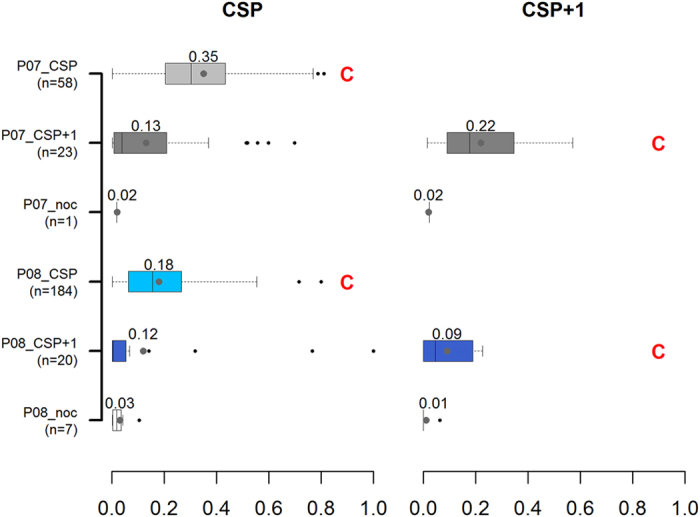
Distribution of Bluetongue prevalence over calving seasons. This figure displays the distribution of Bluetongue prevalence over CSP (the calving season of primary infection by Bluetongue, on the left) and CSP+1 (the calving season that follows CSP, on the right) among units, depending on their periods of primary Bluetongue infection and of first cluster detection. *P07_CSP*, *P07_CSP*+*1* and *P07_noc* units refer to units first infected by Bluetongue in 2007 in which a cluster was detected over CSP, CSP+1, or in which no cluster was detected, respectively. *P08_CSP*, *P08_CSP*+*1* and *P08_noc* units refer to units first infected by Bluetongue in 2008 in which a cluster was detected over CSP, CSP+1, or in which no cluster was detected, respectively. Calving seasons over which a cluster was detected are identified by a red letter C. Numbers refer to average Bluetongue prevalence.

**Figure 6:**
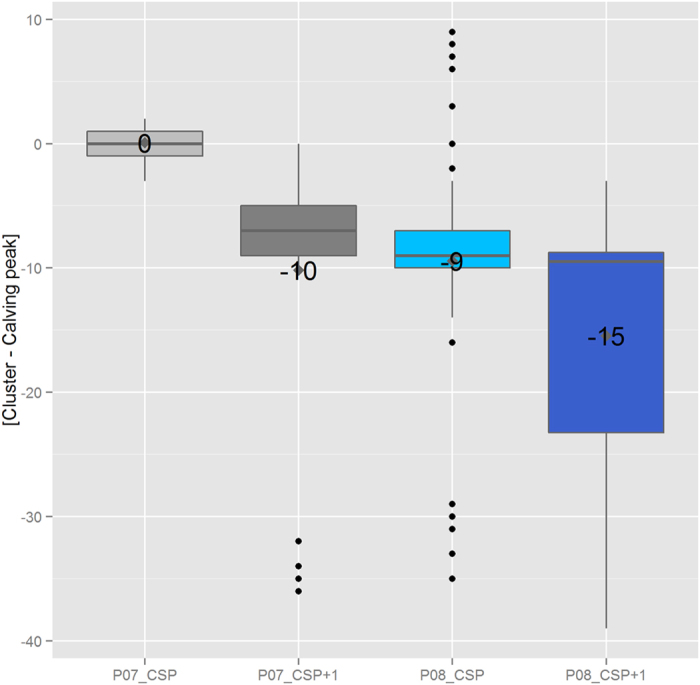
Distribution of the time elapsed between the first cluster detection and the related calving peak. This figure displays the distribution of the time, in weeks, elapsed between the detection of the first cluster in a unit, and the calving peak of the calving season over which this cluster was detected. Units were classified according to the date of first Bluetongue infection and first cluster detection. *P07_CSP*, *P07_CSP*+*1* units refer to units first infected by Bluetongue in 2007 in which a cluster was detected over CSP and CSP+1. *P08_CSP* and *P08_CSP*+*1* units refer to units first infected by Bluetongue in 2008 in which a cluster was detected over CSP and CSP+1. Numbers refer to the average period (in weeks).

Bluetongue cases were reported during every calving season over which a cluster was detected except for seven units, first infected by Bluetongue in 2008 and included in a cluster in CSP+1. Among calving seasons over which a cluster was detected and during which Bluetongue cases were reported, Bluetongue prevalence was significantly higher in the units first infected in 2007 and included in a cluster during their CSP than in others (Fig. 5, boxplots followed by the C red letter) (p-value < 0.0009). The time elapsed between the start of the calving season and the first Bluetongue case (Fig. 4, boxplots followed by the C red letter) was significantly higher in units first infected in 2007 and included in a cluster during their CSP than in others (p-value < 2.10^−6^). The time elapsed between the first Bluetongue case over the calving season and the first cluster detection was significantly higher in units first infected in 2007 and included in a cluster during their CSP than in units first infected in 2008 and included in a cluster during their CSP (mean = 30 and 25 weeks respectively, p-value = 0.01). Lastly, the time elapsed between calving peak and the first cluster detection (Fig. 6) was significantly higher in units first infected in 2007 and included in a cluster during their CSP than in others (p-value < 2.10^−6^).

## Discussion

Using calving data, we mimicked a prospective analysis of spatio-temporal variations of the number of calvings in beef cows among 300 iso-populated units, during the Bluetongue epizootic in France in 2007-2008. Results were analyzed at the unit level, based on clinical Bluetongue cases and calving seasons. Only 1% of clusters were unlikely to be related to Bluetongue. Clusters were detected during the calving season over which the Bluetongue infection appeared (i.e. calving season of primary infection, CSP) in 28% (n = 23) of the units first infected by Bluetongue in 2007, and in 87% (n = 184) of the units first infected by Bluetongue in 2008. In units in which a first cluster was detected over their CSP, Bluetongue was detected more rapidly after the start of the calving season (mean = 10 and 6 weeks in units first infected by Bluetongue in 2007 and 2008, respectively) and Bluetongue prevalence was higher (mean = 35% and 18% in units first infected by Bluetongue in 2007 and 2008, respectively) than in other units which were not included in a cluster over their CSP. Clusters were detected later in the 23 units first infected by Bluetongue in 2007 than in the 184 units first infected by Bluetongue in 2008. Indeed, in the former category of units, clusters were detected on average 30 weeks after the first Bluetongue case and the same week as the calving peak; in the latter category of units, clusters were detected on average 25 weeks after the first Bluetongue case and 9 weeks prior to calving peak.

Our study has some limitations. We identified drops in calvings in most units infected by Bluetongue in 2007 and 2008. Due to the study design, some units in which the number of calvings did not actually drop may have been included in a cluster just because they were inside the cylindrical window in which a significant spatio-temporal cluster of drops in calvings was identified. However, such an analysis provided an in-depth understanding of the relationships between cluster detection, Bluetongue occurrence and calving seasons. We used the Bluetongue epizootic as a case study (an abortive event for which data were available) and it would be interesting to test our method with other abortive events than Bluetongue. Clinical Bluetongue cases were likely to be under-reported^15^,^16^, and Bluetongue prevalence under-estimated. This under-reporting might explain the reason why, although Bluetongue occurred earlier in units first infected in 2008, Bluetongue prevalence was lower than in units first infected in 2007: these contradictory results may be explained by the fact that, in 2008, the relevance of vaccinating cattle against Bluetongue was debated, and conflicts between farmers and vets may have discouraged farmers to notify^1^. But considering the magnitude of the Bluetongue epizootic, exhaustive knowledge of clinical Bluetongue cases would certainly not have greatly modified our results. It could have been of interest to study the influence of vaccination on cluster detection. Indeed, a mass vaccination campaign against Bluetongue was carried out in 2008. Unfortunately vaccination data were not available at the herd level. A slight reduction of fertility was found to be associated with vaccination in a previous study^17^. However, the vaccination effect was low compared to the effect of Bluetongue serotype 8 exposure on fertility and thus, vaccination certainly has little influenced cluster detection^17^. To facilitate the analysis of the results in the light of Bluetongue infection, we adopted our own definition of a calving season. There is a degree of arbitrariness in it, and in some units, the CSP was defined based on a single reported clinical Bluetongue case. Accordingly, the proportion of units included in a cluster over their CSP was slightly underestimated.

Despite these limitations, the approach presented in this paper can be used for other diseases, and provides a useful tool to develop syndromic surveillance systems. By taking into account spatial correlations of variations of the indicator amongst units, it avoids “false” alarms due to isolated events, unlikely to be of interest for veterinary services. Besides, the territory was partitioned into iso-populated units: thus, the ability to detect an abnormal variation of the indicator was homogenized amongst units.

Based on our study, prospective syndromic surveillance using calving data would have made it possible to detect a spatio-temporal cluster of calving drops several weeks after the first clinical Bluetongue cases were recorded. Overall, the ability for this type of syndromic surveillance to detect an abortive event depends on the different factors developed hereafter.

First, our study focused on beef cows only. There is a need to use the same approach to assess the ability for a syndromic surveillance based on calving data to detect abortive events among other cattle populations. This type of syndromic surveillance system based on calving data would cover the entire cattle population at risk in France, with the exception of cows culled following an abortion without any calving notification. Thus, the occurrence of an abortive event, followed by an increase in aborting cows sent to the abattoir, might be undetected or its impact underestimated.

Second, temporal and spatial scales must be adapted to the targeted abortive disease. In our study, variations of calving data were analyzed in units with an average population size of 443 beef cattle farms. This spatial scale was large enough to detect Bluetongue, an abortive event with low within-herd prevalence, but high herd prevalence. It would certainly enable to detect other diseases with the same epidemiological pattern, such as non-infectious diseases (intoxications or metabolic disorders^5^,^18^,^19^,^20^). However, the spatial scale should be smaller, in order to avoid any dilution effects and to rapidly detect an abortive disease with high within-herd prevalence (such as Rift Valley Fever). But regardless of the spatial scale, a disease outbreak with sporadic abortions, such as the brucellosis outbreak detected in France in Haute-Savoie in 2012^18^, would certainly not be detected by our indicator.

Third, the ability to detect an effect if it actually exists, *i.e.* the statistical power of the study, is influenced by the population size and the magnitude of the effect within the spatial units. Consistently, drops in calvings are more likely detected at the time of calving peak. The magnitude of the effect is influenced by the level and duration of exposure, and by the receptivity and susceptibility of the cattle to the abortive disease.

This study showed that the ability to detect a drop in calvings over a calving season was higher in units in which Bluetongue prevalence was high and when Bluetongue cases were reported early in the calving season. Indeed, cows received more exposure to Bluetongue and the risk of reproductive failure was higher^12^. Units first infected in 2007 and included in a cluster during their CSP were located in the north-east of France, where Bluetongue was first introduced in 2007 and impacted cattle herds heavily. In 2008, Bluetongue re-infected all the units first infected in 2007, and some more heavily than in the preceding year; accordingly, these units were included in a cluster during their CSP+1. Besides, Bluetongue progressed westward and southward, and infected new units which were included in a cluster during their CSP.

Likewise, most drops in calvings were identified in winter, after the spread of Bluetongue during the *Culicoïdes* activity period, in the summer and autumn of 2007 and 2008. Cows that calved at this time were exposed during all or most of their pregnancy to Bluetongue and thus were more likely to abort than others. Based on our study, a cluster was detected only if the exposure level was high (higher than 35% on average) and the exposure duration long (on average 30 weeks). But as the risk of reproductive failure in cases of Bluetongue infection is quite low in comparison with other abortive diseases such as brucellosis or Rift Valley Fever, detecting an abortive event is likely to be facilitated in cases with high abortion rates.

Lastly, the number of calvings is an indirect indicator of bovine abortion and lacks specificity. Indeed, a drop in calvings can be due to an increase in reproductive failure occurring at any time during pregnancy, or to an increase in stillbirth or calf mortality within the week after birth. In our study, drop in calvings might have been due to one of these factors, as Bluetongue can cause reproductive failure all throughout pregnancy^12^,^19^. In addition, bovine abortion is an indirect indicator of Bluetongue and lacks specificity. Mimicking a prospective analysis of calving data only made it possible to identify drop in calvings but not to identify the reasons for it. However, to our knowledge, Bluetongue was the only significant abortive event that occurred at the national level from 2007 to 2010. In addition, these results are in accordance with previous studies that revealed the influence of Bluetongue on abortion occurrence^12^,^20^. Based on our study, the number of “false” positive clusters (clusters unlikely to be related to Bluetongue) was low. Of course, it is possible that the high impact of Bluetongue prevents an in-depth analysis of specificity. Moreover, these clusters not due to Bluetongue might be of interest for stakeholders since they reveal that another event had a significant impact on calvings. Clusters were detected faster in units first infected in 2008 than in units first infected in 2007, certainly due to the fact that Bluetongue occurred earlier in 2008 than in 2007 and therefore herds were exposed to Bluetongue longer. Beyond the influence of the exposure level, an abortive disease is likely to be rapidly detected if it causes late abortions (as brucellosis does).

Based on our study, a syndromic surveillance system based on calving data could complement the existing mandatory abortion surveillance system. Further studies are needed to thoroughly investigate the performance of the algorithm under alternative disease pattern scenarios^21^. However, this type of syndromic surveillance can already be implemented, with no additional workload for data providers. It will cover beef cattle, a population in which abortion surveillance is currently particularly weak. Such a surveillance system should contribute to detecting events that would otherwise not have been detected by the current event-driven surveillance, such as a slight increase in abortion occurrence over large areas^22^,^23^, which is unlikely to be reported by farmers due to the low within-herd prevalence of abortions. Under certain circumstances, events could be detected earlier than by the event-driven brucellosis surveillance system. Lastly, even if an event is detected late, the syndromic surveillance system could be used to assess a health impact or the absence of an impact of potentially health-threatening incidents^21^.

## Materials and Methods

### Data

#### Data management at the municipality level

Demographic data and clinical Bluetongue cases are recorded at the municipality level, the smallest French administrative unit. Demographic data were extracted from the French National Cattle Register. They included cattle farm identification numbers, animal characteristics (identification number, birth date, sex and breed), and animal movements (herd identification number, date, reason for entry [birth or purchase]). Data on clinical Bluetongue cases from 2007 to 2009 in France were provided by the French Ministry of Agriculture. During the Bluetongue epizootic, each cattle owner was asked to report every clinically suspect case to his veterinarian, who sampled the suspected animal for confirmation. Data included the farm identification number and date of confirmation of each Bluetongue case, which was used as a proxy of the date of exposure of herds.

The study focused on parous beef cows which have already calved at least once, and ran from 1 August, 2004 to 31 July 2010 (313 weeks). Throughout this article, “cows” shall be used to refer to “parous beef cows”. Data were aggregated on a weekly basis to avoid the weekday effect in the modeling process. For each municipality and each week *w* of the study period, the number of calvings among cows for which a previous calving was notified was calculated. Calves that died within the week after being born were excluded. In addition, the number of cow-days (i.e. the “population at risk”) was calculated as the sum of the number of cows having calved for the last time more than 300 days ago before each day *d* of week *w*. We considered 300 days to be the minimum length of a calving interval. Over the entire study period, the size of the cattle population in each municipality was calculated as the average weekly number of cow-days likely to calve, computed as the sum of the number of cow-days likely to calve over each week of the study period divided by 313.

#### Data aggregation into iso-populated units

The detection of a drop in the number of calvings was based on the difference between the observed and expected number of calvings, for which the level of precision depended on the size of the underlying population. However, as detailed below, scan statistics methods, when used to detect spatio-temporal clusters, do not consider the underlying population. Iso-populated units therefore were used to ensure similar levels of uncertainty among units and therefore a similar ability to detect a difference between the observed and expected number of calvings. We partitioned mainland France into 300 iso-populated units, by aggregating the population sizes of municipalities to obtain almost the same average weekly number of cow-days per unit^24^. Indeed, the ability to detect abnormal temporal variations of the number of calvings during the Bluetongue epizootic was confirmed in a previous study at the *département* level^25^ (there are 100 *départements* in mainland France). Here, we intended to study the feasibility of a syndromic surveillance system at a lower scale than the *département*, to detect as early as possible Bluetongue. However spatial scale had to be large enough, because analysis on a small population shows higher variability, which would lower the performance of the statistical detection methods^26^. We used the algorithm developed by^27^ and implemented in the R Bard package^28^.

For each unit, a population weighted centroid was computed, according to the population size of each municipality it includes. Data recorded for each municipality were aggregated at unit level. As a result, for each unit *i* and each week *w* of the study period, the following data were computed: the number of calvings among cows for which a previous calving was notified (Obs_iw_), the number of cow-days (N_iw_), and the number of clinical Bluetongue cases. The study focused on units for which N_iw_ was not null over each week *w* of the study period.

### Spatio-temporal analysis of drops in calvings

#### Estimation of the expected number of calvings per unit

The difference between the observed (Obs_iw_) and expected (Exp_iw_) number of calvings per unit was analyzed from 1 January 2007 to 1 August 2010, a period over which Bluetongue spread across France. The predictions were derived from a model calibrated for each unit from 1 August 2004 to 31 December 2006. For each unit, three Poisson regression models with over-dispersion were tested over this calibration period, successively including: (1) a linear trend with annual periodicity, (2) a linear trend with annual and six-month periodicities, and (3) a linear trend with annual, six-month and three-month periodicities. To reduce the effect of outliers on the estimate of covariate coefficients, a second round of estimations was performed, weighting the observations by the inverse of their residuals, as proposed by Farrington *et al.*^29^. The most complete model included time covariates as follows:


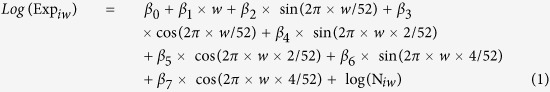


where, N_iw_ is the number of cow-days in unit *i* over week *w* and β the covariate coefficients. The model with the lowest quasi-AIC criterion (QAIC) was chosen^30^,^31^.

#### Analysis of the difference between observed and expected numbers of calvings

Spatio-temporal clusters of drops in the number of calvings were sought using a prospective space-time scan statistic implemented in SaTScan^32^, on a weekly basis^33^. Prospective analysis differs from retrospective space-time scan statistic: it relies on repeated time periodic analyses whereas retrospective space-time scan statistic tests in one shot whether a disease is randomly distributed over space and time for a predefined geographical region during a predetermined time period. For a week *w*_*c*_, clusters were detected using cylindrical risk windows whose radius varied from zero (i.e. only one unit) to 5% of the total number of units. Height represented time and varied from zero (i.e. only one week *w*_*c*_) to 8 weeks (i.e. weeks from *w*_*c*_−*8* to *w*_*c*_). The upper bounds of radius and height of the cylindrical risk window were chosen to ensure early detection of a drop in calvings.

The prospective space-time scan statistic was run for each week w_c_ included in the period from 26 February 2007 (to enable cluster detection up to 8 weeks prior to week *w*_*c*_) to 31 July 2010. For each week *w*_*c*_, the model input was the observed number of calvings Obs_iw_ and the expected number of calvings Exp_iw_, available for each unit *i* and each week *w* from 1 January 2007 to week *w*_*c*_. Under the null hypothesis, the ratio of the observed number of calvings to the expected number of calvings inside cylindrical risk window Z was not significantly different from the same ratio calculated outside the risk window. More precisely, the number of calvings observed inside cylindrical risk window Z followed a Poisson distribution with a mean equal to:


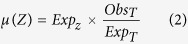


where Obs_T_ was the total number of calvings observed among all units over the period from 1 January 2007 to week *w*_*c*_, Exp_T_ the total number of calvings expected among all units and over the period from 1 January 2007 to week *w*_*c*_, and Exp_Z_ the number of calvings expected inside cylindrical risk window Z. The value of the likelihood ratio (LLR) was calculated for every possible cylinder^10^. The scan statistic method used prospectively has been criticized due to lack of control of the type-I error probability^34^. Therefore, we selected clusters based on LLR (with an LLR above a specified threshold) rather than on the p-value^11^.

### Detection of clusters of drops in calvings

#### Definition of calving seasons at the unit level

For each unit, a calving peak over year Y was defined as the mode of the weekly number of calvings predicted over this year. A calving season was defined as the period starting 3 months after the calving peak of a given year (when most cows were likely to be at the early stage of their pregnancy) and ending 3 months after the calving peak of the following year (once most cows have calved). This allowed the calving season to vary between units to reflect the different practices in place in different regions of France. For each unit, calving seasons were defined from 1 January 2006 to 1 August 2010. The first clinical Bluetongue case reported over the entire study period in a unit was used to define: 1) the first year of infection of the unit by Bluetongue (2007 or 2008); 2) the first calving season over which this first clinical Bluetongue case was detected in the unit, hereafter referred to as calving season of primary infection (CSP). The calving season that follows CSP was named CSP+1 (Fig. 1a,b). The influence of Bluetongue infection on cluster detection was analyzed by considering each calving season separately. Therefore, for each unit and each calving season, we computed the time elapsed between the start of this calving season and the first clinical Bluetongue case, and the Bluetongue prevalence (*i.e*. the ratio of the total number of cattle herds with clinical Bluetongue cases reported over this calving season to the total number of cattle herds, regardless of the production type) (Fig. 1a,b).

#### Cluster selection over calving seasons

For each unit, we identified the first cluster that included it, and which was detected following the first clinical Bluetongue case in the unit. This cluster was assigned to one of the two calving seasons of the unit, CSP or CSP+1 (Fig. 1a,b). Units were split in six categories, depending on the year of their first infection by BT and of the calving season over which the cluster was detected (CSP or CSP+1): units first infected by Bluetongue in 2007 in which the first cluster was detected over CSP, CSP+1, or in which no cluster was detected, and units first infected by Bluetongue in 2008 in which the first cluster was detected over CSP, CSP+1, or in which no cluster was detected.

#### Sensitivity and specificity of cluster detection

A syndromic surveillance system should try to maximize sensitivity and specificity. In order to determine which definition of an alarm was most effective in this regard, we selected an LLR threshold based on 1) the specificity of the surveillance system, by studying the number of false positive clusters (i.e. clusters detected before any clinical Bluetongue case report in any unit it included); 2) the sensitivity of the surveillance system, defined as the proportion of units for which the first cluster was detected over CSP, calculated among units first infected by Bluetongue in 2007 (as the ratio of the number of units first infected in 2007 and included in a cluster during their CSP to the total number of units first infected in 2007), and units first infected by Bluetongue in 2008 (as the ratio of the number of units first infected in 2008 and included in a cluster during their CSP to the total number of units first infected in 2008)

The risk of abortion was expected to increase over the CSP if cows were highly exposed to Bluetongue, i.e. if Bluetongue prevalence is high or if Bluetongue cases occurred early in the CSP. To study the influence of Bluetongue exposure on sensitivity, we modeled the distribution of Bluetongue prevalence over CSP and of the time elapsed between the start of the CSP and its first clinical Bluetongue case both by differentiating units first infected in 2007 or 2008, included in a cluster during their CSP or CSP+1, or not included in a cluster (see Supplementary material).

#### Early cluster detection over a calving season

When syndromic surveillance is conducted prospectively, the aim is to detect the occurrence of a given disease as early as possible. For each unit and each calving season over which a cluster was detected, the time elapsed between the first clinical Bluetongue case in this unit and the first cluster detected within this unit was calculated (Fig. 1a,b). As calving seasonality was likely to influence the time a cluster was detected, the time elapsed between calving peak and first cluster detection was also computed (Fig. 1a,b). We also studied the time elapsed between the beginning of the calving season over which a cluster was detected and the first clinical Bluetongue case, and the Bluetongue prevalence (Fig. 1a,b). The distribution of the three periods and of Bluetongue prevalence among calving seasons over which a cluster was detected were modeled by differentiating units first infected in 2007 or 2008, included in a cluster during their CSP or CSP+1 (see Supplementary material).

All statistical analyses were computed using R^35^.

In conclusion, this study allowed us to assess the ability to detect an increase in abortion occurrence by analyzing spatio-temporal variations in the number of calvings. While it was focused on a specific event, the Bluetongue epizootic, we believe that this type of syndromic surveillance system could improve the surveillance of abortive events in French cattle, and could complement the current mandatory abortion surveillance system.

## Additional Information

**How to cite this article**: Bronner, A. *et al.* Syndromic surveillance of abortions in beef cattle based on the prospective analysis of spatio-temporal variations of calvings. *Sci. Rep.* **5**, 18285; doi: 10.1038/srep18285 (2015).

## Supplementary Material

Supplementary Information
