# Supplementary material for: Syndromic surveillance of abortions in beef cattle based on the prospective analysis of spatio-temporal variations of calvings
Source: Sci Rep. 2015 Dec 21;5:18285. doi: 10.1038/srep18285 (PMC4685302; doi:10.1038/srep18285)
Supplement: Supplementary Information [file srep18285-s1.pdf]

Syndromic surveillance of abortions in beef cattle based on the prospective analysis of spatio-temporal variations of calvings

A. Bronner[1], E. Morignat[1], G. Fournié[2], T. Vergne[2], J-L Vinard[1], E. Gay[1], D. Calavas[1*]

[1]ANSES-Lyon, Unité Epidémiologie, Lyon, France

[2] Royal Veterinary College, Hatfield, Hertfordshire, UK

[*]Corresponding author

Email addresses:

AB : anne.bronner@anses.fr

EM : eric.morignat@anses.fr

TV : tvergne@rvc.ac.uk

GF : gfournie@rvc.ac.uk

JLV : jean-luc.vinard@anses.fr

EG : emilie.gay@anses.fr

DC : didier.calavas@anses.fr

Supplementary material

Models used to study the influence of Bluetongue exposure on the sensitivity, the specificity and early cluster detection

BT refers to Bluetongue. *CSP* refers to the first calving season over which this first clinical Bluetongue case was detected in the unit, and *CSP+1* to the calving season that follows *CSP*. *P07_CSP*, *P07_CSP+1* and *P07_noc* units refer to units first infected by Bluetongue in 2007 in which a cluster was detected over CSP, CSP+1, or in which no cluster was detected, respectively. *P08_CSP*, *P08_CSP+1* and *P08_noc* units refer to units first infected by Bluetongue in 2008 in which a cluster was detected over CSP, CSP+1, or in which no cluster was detected, respectively.

Sensitivity and specificity

The influence of BT prevalence over CSP ($Prev_{CSP}$) on the sensitivity of cluster detection was studied by running the following logistic regression models:

$$Logit\left(Prev_{CSP,i}\right) = \beta_0 + \beta_1 \times Units_{P07,i} \qquad Logit\left(Prev_{CSP,i}\right) = \beta_0 + \beta_1 \times Units_{P08,i}$$

The influence of the time elapsed between the start of the CSP and its first clinical BT case ($[Start - BT]$) on the sensitivity of cluster detection was studied by running the following linear Gaussian models:

$$[Start - BT]_i = \beta_0 + \beta_1 \times Units_{P07,i} \qquad [Start - BT]_i = \beta_0 + \beta_1 \times Units_{P08,i}$$

Each model included a categorical covariate, $Units_{P07}$ (to differentiate *P07_CSP* -the reference-, *P07_CSP+1* and *P07_noc*) or $Units_{P08}$ (to differentiate *P08_CSP* –the reference-, *P08_CSP+1* and *P08_noc* units).

Early cluster detection over a calving season

The time elapsed between the first clinical BT case and the first cluster detection ($[BT - Cluster]$), between calving peak and the first cluster detection ($[Cluster - Calving\ Peak]$), and between the start of the calving season over which a cluster was detected and the first clinical BT case ($[Start - BT]$) were studied by running the following linear Gaussian models:

$[BT - Cluster]_i = \beta_0 + \beta_1 \times Units_{Cluster,i}$

$[Cluster - Calving\ Peak]_i = \beta_0 + \beta_1 \times Units_{Cluster,i}$

$[Star - BT]_i = \beta_0 [Start - BT]_i = \beta_0 + \beta_1 \times Units_{Cluster,i}$

The distribution of BT prevalence among calving seasons over which a cluster was detected ($Prev_{Cluster}$) was studied by running the following logistic regression model:

$Logit\left(Prev_{Cluster,i}\right) = \beta_0 + \beta_1 \times Units_{Cluster,i}$

Each model included a categorical covariate $Units_{cluster}$ to differentiate *P07_CSP* units (the reference), *P07_CSP+1*, *P08_CSP* and *P08_CSP+1* units.
